# Robust control of electrohydraulic soft robots

**DOI:** 10.3389/frobt.2024.1333837

**Published:** 2024-08-02

**Authors:** Angella Volchko, Shane K. Mitchell, Tyler G. Scripps, Zoe Turin, J. Sean Humbert

**Affiliations:** ^1^ Paul M. Rady Department of Mechanical Engineering, University of Colorado Boulder, Boulder, CO, United States; ^2^ Artimus Robotics Inc., Boulder, CO, United States

**Keywords:** robust control, soft robotics, linear system theory, H infinity synthesis, uncertainty analysis, HASEL actuators

## Abstract

This article introduces a model-based robust control framework for electrohydraulic soft robots. The methods presented herein exploit linear system control theory as it applies to a nonlinear soft robotic system. We employ dynamic mode decomposition with control (DMDc) to create appropriate linear models from real-world measurements. We build on the theory by developing linear models in various operational regions of the system to result in a collection of linear plants used in uncertainty analysis. To complement the uncertainty analyses, we utilize $H_{\infty }$ (“H Infinity”) synthesis techniques to determine an optimal controller to meet performance requirements for the nominal plant. Following this methodology, we demonstrate robust control over a multi-input multi-output (MIMO) hydraulically amplified self-healing electrostatic (HASEL)-actuated system. The simplifications in the proposed framework help address the inherent uncertainties and complexities of compliant robots, providing a flexible approach for real-time control of soft robotic systems in real-world applications.

## 1 Introduction

Soft robotic systems are under constant development and refinement as they offer advantages over conventional rigid robotic solutions. With their unrivaled potential to navigate unstructured environments, enable safe human-robot interactions, and interact with delicate items, soft robots could offer improvements in various real-world applications, including advancements in surgical devices, search and rescue efforts, and industrial automation (Kim et al., 2013; Rus and Tolley, 2015; Majidi, 2019; El-Atab et al., 2020). Several types of soft actuators have been developed in recent years as discussed in the survey, El-Atab et al. (2020), including those that rely on electrostatic actuation (Pelrine et al., 2000; Gu et al., 2017), those that use electrohydraulic actuation (Acome et al., 2018), those that respond to heat (Du et al., 2023), and those that are driven by pressure (Walker et al., 2020). A wide range of sensors have recently been innovated for use in soft systems as detailed in the review paper, Hegde et al. (2023), including resistive sensors (Bilodeau et al., 2015), capacitive sensors (Nguyen et al., 2019), optic sensors (Zhao et al., 2016), and magnetic sensors (Sundaram et al., 2023). While various soft technologies have emerged in recent years, the infrastructure and standardized framework surrounding model development and controller synthesis for these systems is deficient. The success of soft robotic systems implemented in the real-world depends on the realization of effective real-time controllers (Thuruthel et al., 2018).

Given the proliferation of these individual soft technologies, it has become increasingly difficult to determine a standardized process for developing models and control laws for the conglomerative soft systems. There is tremendous variability just within the slew of available sensors and actuators. In addition to the countless soft physical systems that could be conceived, the individual technologies are still undergoing iterative design processes, and are not yet ready to be mass produced (Lipson, 2014). This implies that there is potential for manufacturing inconsistencies from unit to unit. Additional physical unpredictabilities found in these systems could be due to the nature of their soft material properties. After accounting for the manufacturing variability, soft robotic systems can also possess highly nonlinear dynamics and nearly infinite degrees of freedom (Thuruthel et al., 2018). While rigid robots have the luxury of predictability and standardization, all of the aforementioned variables affect the overall dynamics and uncertainties associated with soft robots.

This paper presents a novel approach to model and control soft robotic systems in a structured, repeatable, and robust manner, accounting for their inherent uncertainties and complexities. This study demonstrates the first implementation of an $H_{\infty }$ control law on an electrohydraulically-actuated soft robotic system. Additionally, we build upon previous work in this area by conducting uncertainty analyses on the closed loop systems to ensure their robustness. To maintain a feasible controller that works with various combinations of soft-actuated and soft-sensed robots, we emphasize the importance and art of simplicity as it relates to modelling and control design. The notion of simplicity in this article is three-fold.

First, we rely on data-driven methods to alleviate the burden of deriving dynamical models via first principles for these complex systems. Modelling plays a substantial role in any controller synthesis process, and it is critical that the model obtained is reasonable and accurate to synthesize an appropriate controller. Given the current developmental state of soft robots, their potentially unpredictable mechanical behavior, and their innate nonlinearities, data driven modelling techiques are efficient tools for soft system identification. Further, empirical methods eliminate the need to rederive the complex dynamics from first principles with each system. Specifically, we discuss the data driven reduced order modelling system identification technique, dynamic mode decomposition with control (DMDc) (Proctor et al., 2016).

Our second simplification is to employ linearization as a means of modelling the nonlinear, possibly infinite dimensional systems. We desire the linear model to be an appropriate approximation of the robot’s dynamics. While it is important that the model represents the real-world system, we exploit a concept from linear system theory that the linearizing effects created by feedback control allow us to relax the accuracy of the models and allow for larger deviations from steady state (Skogestad and Postlethwaite, 2005). DMDc produces this desired linear model from empirical data.

Lastly, to complement the linear controller development, we employ robust control theory that enables us to analyze the system’s uncertainty and determine the success of the controller across the system’s operating range. The robustness analysis can be extended to determine if the system remains stable in the presence of inconsistencies due to manufacturing processes in addition to the neglected or unmodelled dynamics. To simplify the numerous potential sources of error from these systems, we “lump the uncertainty” into a single complex perturbation (Skogestad and Postlethwaite, 2005). We demonstrate the simplicity and efficacy of this generalized framework through the development of a controller for a nonlinear soft robotic system driven by HASEL actuators.

**FIGURE 1:**
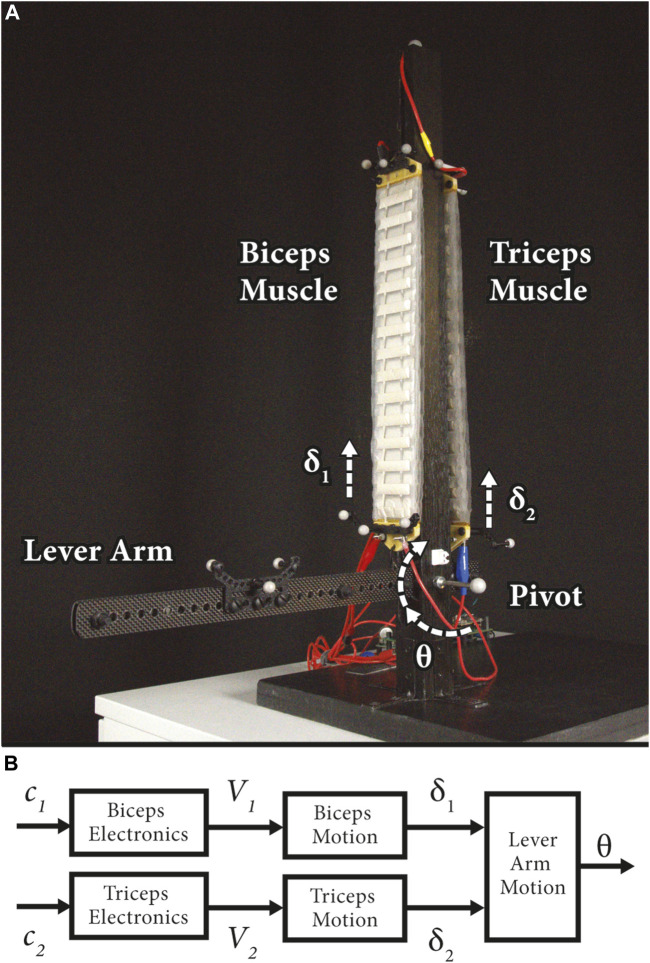
**(A)** The benchtop system emulates the upper portion of the human arm, where the antagonist muscles, biceps and triceps, work together to control the orientation, θ , of the lever arm around the pivot. The experimental setup consists of two stacks of HASEL actuators attached to the lever arm on either side of the vertical post. Each artificial muscle consists of five 15-pouch Peano-HASEL actuators that contract by δ on actuation. The motion capture system measures the position of the motion capture rigid bodies placed throughout the system. Rigid bodies were attached to both ends of each stack of HASELs to measure the contraction of the actuator, δ . Another rigid body was secured to the lever arm, to capture the orientation of the system, θ . **(B)** This diagram describes the signals sent within the system and controlled in this work. The lowest level signals, charge (c) , are sent through the HASEL electronics. These signals determine the voltages, V , of each individual actuator which directly affects the displacement, δ , of each actuator. Lastly, the contraction of the actuators works to control or steer the overall orientation, θ , of the lever arm.

The system, depicted in Figure 1A, emulates the biceps and triceps configuration of the upper portion of the human arm using two antagonist stacks of contracting HASEL actuators. HASEL actuators are an optimal actuator technology for testing the presented modelling and controller synthesis framework due to their precision (Volchko et al., 2022), muscle-like performance (Rothemund et al., 2021), and fast dynamics (Kellaris et al., 2018; Mitchell et al., 2019). In this work, Peano-HASEL actuators were used due to their linear contraction on activation (Kellaris et al., 2018), which enables bioinspired robotic designs. While modelling and closed loop efforts have been made in the world of HASEL actuators (Schunk et al., 2018; Johnson et al., 2020; Rothemund et al., 2020; Volchko et al., 2022), none of them have demonstrated the application of robust control techniques.

The generalized linear system and robust control framework demonstrated in this paper could facilitate fast real-time control of soft robotic systems in real-world applications. In particular, we could apply this framework to electrohydraulic actuators used to control pumps or valves for fluid metering (Fuaad et al., 2023). In such systems, it would be important to adopt robust position control laws to help ensure accurate fluid flow and dosing. This framework could also be applied to bio-inspired swimming robots that rely on electrohydraulic actuation (Hess and Musgrave, 2023). These aquatic systems could benefit from robust control laws that increase their efficiency and maneuverability in hydrodynamic exploration of various underwater environments—aiding in CO_2_ monitoring and climate change predictions, for example,.

This paper is comprised of the following sections. Section 2 introduces the theory that is pivotal to the presented framework, including (2.1) an overview of linear system control theory, (2.2) multivariable robust control concepts, and (2.3) dynamic mode decomposition. Section 3 discusses the innerworkings of the HASEL actuator along with its advantages and relevance to this work. The subsequent Section 4 describes the experimental benchtop system, the methods applied, and the resultant performance. Two subsections describe the modelling and controller implementation of both the voltage dynamics (4.1) and the displacement dynamics (4.2). Finally, we conclude with discussion and future recommendations in Section 5.

## 2 Theory

This section aims to situate the experimental and applied work described in Section 4 within the broader framework outlined in this paper. By providing background information on the theory, readers can gain perspective on the scope of this methodology, understand its limitations and potential applications, and further develop this research for their own studies. The equations described in this section provide insight and context for the figures and plots presented in this work.

### 2.1 Linear system control theory

At first glance, it may seem unintuitive to apply linear system control theory to nonlinear soft robotic systems. However, it has been demonstrated that linear modelling and control techniques have been successful in enabling real-time control of other nonlinear dynamical systems working in real-world situations, such as helicopter flight control (Smerlas et al., 2001) and hydraulic actuator control (Niksefat and Sepehri, 2001). Implementing feedback control creates a local linearizing effect in which the linear model remains valid since the negative feedback loop keeps the system output near the desired state. This justifies the use of linear models in control design of nonlinear systems (Skogestad and Postlethwaite, 2005). Additionally, there is a global linearizing effect since the reference to output response is approximately linear for stable closed-loop systems (Skogestad and Postlethwaite, 2005). Many readily available and intuitive controller synthesis and analysis tools are designed for linear systems. Linear system theory provides us with a road map to create feasible control laws that can guarantee performance requirements, even in the presence of uncertainty. Further detail on the following overview of this theory can be found in (Chen, 1984; Skogestad and Postlethwaite, 2005).

Many physical dynamical systems can be described with the following set of equations:

$$\dot{x}\left(t\right)=f\left(x\left(t\right),u\left(t\right)\right)$$ (1)

$$y\left(t\right)=g\left(x\left(t\right),u\left(t\right)\right)$$ (2)

where x represents the state of the system at time, t , u represents the input to the system, y represents the output of the system, $\dot{x}$ is the time derivative of x , and f and g are any nonlinear functions. The models developed within this research are limited to the scope of finite linear time-invariant (LTI) models. Thus, this work uses the following linear description, or state-space form:

$$\dot{x}\left(t\right)=Ax\left(t\right)+Bu\left(t\right)$$ (3)

$$y\left(t\right)=Cx\left(t\right)+Du\left(t\right),$$ (4)

with signals x ϵ $R^{n}$ , u ϵ $R^{m}$ , y ϵ $R^{l}$ , and $\dot{x}$ ϵ $R^{n}$ . The matrices A ϵ $R^{n\times n}$ , $B\;\epsilon \;R^{n\times m}$ , $C\;\epsilon \;R^{l\times n}$ , and $D\;\epsilon \;R^{l\times m}$ correspond to the state, input, output, and feedthrough matrices, respectively.

While it is convenient to demonstrate performance metrics and analyze uncertainty in continuous time, it is necessary to collect data and implement digital controllers in discrete time. We denote the discrete-time LTI systems as:

$$x_{k+1}=\bar{A}x_{k}+\bar{B}u_{k}$$ (5)

$$y_{k}=\bar{C}x_{k}+\bar{D}u_{k},$$ (6)

where kϵR represents a discrete time step, kΔt . Representing systems in the frequency domain enables quantitative and qualitative analysis by leveraging analytical tools such as Bode plots and singular value plots. To translate time domain models into their frequency domain counterparts, we use the Laplace transform to shift the model’s dependence from time, t ϵ R , to the complex frequency-domain parameter, s=α+jω , where αϵR , jϵC , and frequency, ωϵR . Throughout this work, we demonstrate controller synthesis techniques as they apply to both single-input single-output (SISO) and multi-input multi-output (MIMO) dynamical systems, which can both be represented by Eqs 3–6. Using the matrices from Eqs 3, 4, we can describe the plant transfer function,

$$G\left(s\right)=C{\left(sI-A\right)}^{-1}B+D,$$ (7)

where the identity matrix $I\;\epsilon \;R^{n\times n}$ . The goal of controller synthesis is to systematically design a controller to change the behavior of the system in order to achieve nominal performance, meaning that the system satisfies performance specifications for the nominal plant. Ideally, we would directly tune the closed loop system response, however this transfer function has a nonlinear dependence on the controller or an indirect relation with the controller gains. We instead modify the open loop response and relate this to our closed loop performance specifications following the loop shaping methodology described in Åström and Murray (2021). We can apply this approach using Bode plots to visualize the magnitude and phase of the open loop transfer function of a system,

$$L\left(s\right)=G\left(s\right)K\left(s\right),$$ (8)

as a function of frequency to synthesize a linear controller, K(s) , for the plant, G(s) . Loop shaping provides “design knobs” within K(s) to make the closed loop system,

$$T\left(s\right)=L\left(s\right){\left(I+L\left(s\right)\right)}^{-1},$$ (9)

meet performance specifications. These design knobs are used to intuitively manipulate the shape of |L(jω)| as any adjustments to the controller are directly related to the magnitude of |L(jω)| , which we can then relate to the closed loop behavior of T(s) . While worst case signal input to output gains for a SISO system can be represented with conventional input to output Bode plots, it is important that MIMO system analyses rely on singular value Bode plots. Since MIMO systems introduce coupling between system states, maximum singular values, $\bar{\sigma }$ , are required to capture the worst-case signal amplifications. The maximum singular value of G(s) is:

$$\bar{\sigma }\left(G\left(j\omega \right)\right)=\underset{w\left(\omega \right)\neq 0}{\max}\frac{{\left\|z\left(\omega \right)\right\|}_{2}}{{\left\|w\left(\omega \right)\right\|}_{2}},$$ (10)

where w(ω) is a sinusoidal input to the system at a given frequency, ω , z(ω) is the output, and ${\left\|\cdot \right\|}_{2}$ is the 2-norm:

$${\left\|z\left(\omega \right)\right\|}_{2}=\sqrt{\underset{i}{\sum }|z_{i}\left(\omega \right){|}^{2}}=\sqrt{z_{1}{\left(\omega \right)}^{2}+z_{2}{\left(\omega \right)}^{2}+\cdots +z_{n}{\left(\omega \right)}^{2}}$$ (11)

The signal gains captured by these singular values correspond directly to the $H_{\infty }$ norm of the system, which is the maximum singular value over all frequencies,

$${\left\|G\left(s\right)\right\|}_{\infty }\;≜\;\underset{\omega }{\sup}\bar{\sigma }\left(G\left(j\omega \right)\right).$$ (12)

The $H_{\infty }$ norm can be used to quantify the magnitude of transfer functions that describe the performance of a system. These transfer functions include the “sensitivity,” S , the product KS , and the “complementary sensitivity,” T . In controller synthesis, S ,

$$S\left(s\right)={\left(I+G\left(s\right)K\left(s\right)\right)}^{-1},$$ (13)

describes the closed loop response from reference signals, $w_{1}$ , to control error signals, ${\tilde{z}}_{1}$ and informs regulation of system response with respect to reference signals, $w_{1}$ , and low frequency external disturbances, $w_{3}$ . We modify S to optimize for reference tracking, transient behavior, and steady state error. We use the closed loop transfer function KS ,

$$KS\left(s\right)=K\left(s\right){\left(I+G\left(s\right)K\left(s\right)\right)}^{-1},$$ (14)

to describe the control output signal, ${\tilde{z}}_{2}$ , in response to the reference signals. KS provides us with an idea of the control effort required of the system. Lastly, T (Eq. 9) describes the closed loop response from output disturbance signals, $w_{2}$ , to system output, ${\tilde{z}}_{3}$ . This transmission helps us understand the system’s robustness and sensitivity to noise. Optimizing these transfer functions based on the $H_{\infty }$ norm translates into minimizing the peak singular values over the frequency range relevant to the control problem.

**FIGURE 2:**
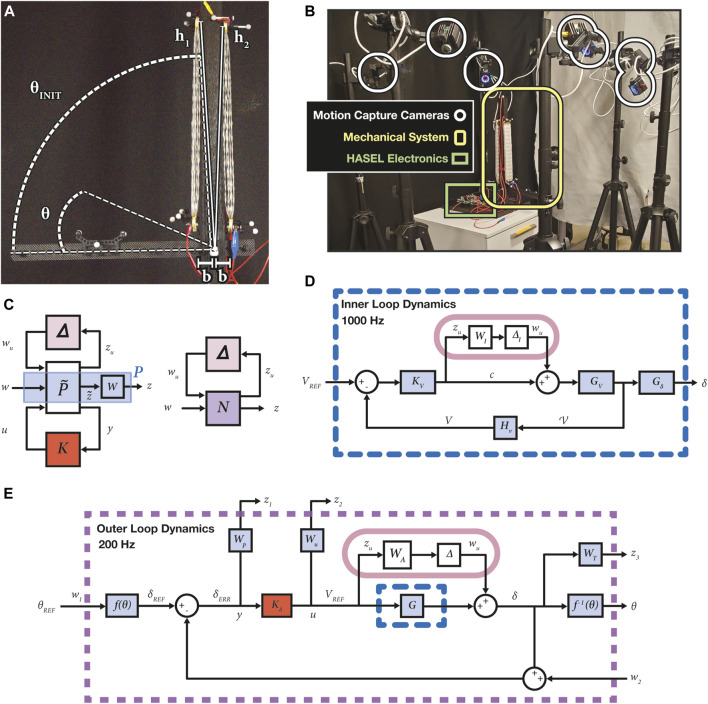
**(A)** The HASEL actuators were secured from the pivot at distances b=33 mm along the lever arm and $h_{1}=393$ mm and $h_{2}=387.3$ mm from above. The actuator length, l=393 mm, was measured between its two anchor points. The angle desired, $\theta_{\text{REF}}$ , was prescribed about the pivot of the lever arm, $\theta_{\text{INIT}}$ . **(B)** The experimental setup consisted of seven OptiTrack Prime13W motion capture cameras surrounding the system to track the retroreflective markers. All other reflective items were mitigated. The HASEL electronics were located on the same platform as the mechanism. They were connected to the wall outlet for power and the Linux computer for ROS. **(C)** The generalized block diagram can be applicable to nearly any linear control problem. It contains the nominal plant, P , the controller, K , and plant uncertainty, Δ . This diagram depicts the basis of $H_{\infty }$ controller synthesis and μ -analysis approaches. The signals represented in this diagram include the plant input signals, w , the plant output signals, $\tilde{z}$ , the weighted output signals, z , the control output signal, u , and the system output signal, y . The weighting functions $W_{P}$ , $W_{U}$ , $W_{T}$ are wrapped up in the block, P . Upon choosing a controller K to minimize the effects of w on z , the lower half of this generalized block diagram can be folded into the lower LFT to create the resultant block N . Following the controller synthesis, the upper half of these diagrams that include the uncertainty input and output signals, $w_{u}$ and $z_{u}$ , respectively, are used to assess the effect of the uncertainty on the closed loop system. **(D)** The inner loop dynamics block diagram depicts all of the signals and transfer functions needed to implement the voltage controller. This SISO loop incorporated a multiplicative model for uncertainty, which is represented with $W_{I}$ and $\Delta_{I}$ . The voltage controller, $K_{V}$ , compared the voltage measured by the voltage monitor, $H_{V}$ , to the reference voltage, $V_{\text{REF}}$ , to create a signal, c , that was sent to the HASEL electronics, $G_{V}$ , which then provided a voltage to the actuators, V , to cause the actuators to contract, $G_{\delta }$ , and affect the overall system displacement, δ . This loop was closed at 1,000 Hz on the microcontroller. **(E)** The cascade control scheme is demonstrated through this block diagram. All of the inner loop dynamics were accounted for within the transfer function, G , denoted with the blue dashed line, corresponding to the blue dashed line in **(D)**. The reference orientation, $\theta_{\text{REF}}$ , was sent through the function, f(θ) , to result in the reference displacements, $\delta_{\text{REF}}$ , which was compared to the current estimated actuator displacements, δ , to result in the displacement errors, $\delta_{\text{ERR}}$ . All of the signals in and out of P were recognized in the system, including the reference signal, $w_{1}$ , the output disturbance, $w_{2}$ , and the weighted outputs, $z_{1}$ , $z_{2}$ , and $z_{3}$ . All performance weightings are also depicted as $W_{P}$ , $W_{U}$ , and $W_{T}$ . The displacement controller is highlighted in red. The outer loop controller was closed at 200 Hz. Following the color coding, this diagram can be rearranged into the structure provided in the generalized block diagram **(C)**.

In order to formulate this optimization problem, the system must be restructured such that all the input signals, w , are grouped together as the input to a single MIMO transfer function, N , demonstrated in Figure 2C, which produces the combined outputs of the system, $\tilde{z}$ . Weights are applied to the outputs to result in $z_{1}$ , $z_{2}$ , and $z_{3}$ and provide “tuning knobs” for controller synthesis. Then N can be optimized. This problem formulation can be applied to almost any linear system (Skogestad and Postlethwaite, 2005).

Individual input signals $w_{1}$ , $w_{2}$ , $w_{3}$ can be collected into a vector-valued signal, w , represented in the generalized block diagram in Figure 2C. Similarly, weighted outputs $z_{1}$ , $z_{2}$ , and $z_{3}$ can be collected and represented as exogenous output signal, z , as the output of the generalized plant model, P (Figure 2C). This generalized depiction represents the open loop plant, P , broken at each of these signals. The presented framework uses this formulation to determine a controller, K , that provides a control signal, u , based on the error signal, v , to minimize the effect of the input signals, w , on the weighted output signals, z . The plant, P , can be divided into

$$P=\left[\begin{array}{cc} P_{11} & P_{12}\\ P_{21} & P_{22} \end{array}\right],$$ (15)

where

$$z=P_{11}w+P_{12}u$$ (16)

$$v=P_{21}w+P_{22}u$$ (17)

and $P_{11}$ , $P_{12}$ , $P_{21}$ , $P_{22}$ are compatible with the dimensions of their respective column vectors, u , v , w , and z . The $H_{\infty }$ weighted sensitivity process begins with selecting the weighted transfer functions, $W_{p}$ , $W_{u}$ and $W_{T}$ , depicted in Figure 2E. These weights work to emphasize different frequency portions of the closed loop transfer functions, S , KS , and T , with respect to one another. They provide “tuning knobs” which can be visualized with their respective singular value plots. The terms in the performance weight, $W_{p}\left(s\right)=diag\left(w_{p_{i}}\right)$ , can be chosen as

$$w_{p_{i}}\left(s\right)=\frac{{\left(s/M^{1/n}+\omega_{b}^{*}\right)}^{n}}{{\left(s+\omega_{b}^{*}A^{1/n}\right)}^{n}},$$ (18)

where real scalars M , $\omega_{b}^{⋆}$ , and A are selected to tune the system’s transient response, including rise time and overshoot, its bandwidth, and its steady state error, respectively. The overall shape of this transfer function also affects tracking and disturbance rejection and can be further tuned by adjusting the scalar, n . Initial elements $w_{u_{i}}$ in $W_{u}$ are selected such that

$$w_{u_{i}}\left(s\right)=\frac{s}{s+\omega_{b}},$$ (19)

where $\omega_{b}$ is approximately the desired closed loop bandwidth. Lastly, elements in $W_{T}$ are adjusted so that $w_{T_{i}}\ll 1$ at low frequencies and $w_{T_{i}}\gg 1$ at high frequencies to reduce sensitivity to noise and uncertainty. These signals are depicted in the outer loop dynamics block diagram (Figure 2E).

After adjusting the weighting functions, we can find the optimal $H_{\infty }$ controller by closing the lower portion of the generalized plant diagram to form the lower linear fractional transformation, N , using the segmented generalized plant, P ,

$$N=P_{11}+P_{12}K{\left(I-P_{22}K\right)}^{-1}P_{21}.$$ (20)

N can be denoted as the stack of sensitivities with their corresponding weights,

$$N=\left[\begin{array}{c} W_{p}S\\ W_{u}KS\\ W_{T}T \end{array}\right],$$ (21)

such that we can minimize its $H_{\infty }$ norm,

$$\gamma_{\min}=\underset{K}{\min}{\left\|N\left(K\left(s\right)\right)\right\|}_{\infty },$$ (22)

where $\gamma_{\min}\;\epsilon \;R$ . Using MATLAB’s Robust Control toolbox, we can solve for this optimization to result in the optimal $H_{\infty }$ controller, K(s) . Further details of this process are described in the methods section. This mixed-sensitivity controller synthesis approach simultaneously optimizes for the three transfer functions in N : $W_{p}S$ , $W_{u}KS$ , and $W_{T}T$ . While we want the resultant $\gamma_{\min}$ to be less than 1 to mathematically satisfy the closed loop specifications, the specifications are rough guidelines for our desired performance, and thus, we iterate our control design within the $H_{\infty }$ optimization framework in order to balance design tradeoffs, as suggested in (Skogestad and Postlethwaite, 2005). Therefore, we treat the terms in these weighting functions as tuning knobs until we achieve satisfactory system performance. While this section provides an overview of the linear system theory essential to the proposed controller synthesis framework, it is crucial to assess the error arising from such linear assumptions in developing models and controllers for nonlinear soft robotic systems.

### 2.2 Robust control theory

Robust control theory promotes the development of controllers that are effective in real world applications due to its ability to assess system properties such as stability and performance in the presence of system uncertainty. Within the robust control domain, we focus our work on robust stability. Robust stability signifies that all plants in a set of possible plants remain stable with the implementation of a selected controller. The uncertainty discussed in this section regards the variation from a set of possible plants to the nominal system model. Stability of a system is critical in real-world implementation and means that for a bounded input signal, the internal signals remain bounded. This translates to the system converging to equilibrium over time. Robust control theory has been applied to a multitude of fields including aerospace systems, chemical processes, power networks and fluidic systems (Dullerud and Paganini, 2013). However, it is not yet prevalent in the field of soft robotics.

Robust control techniques could be beneficial and complementary to the field of soft robotics, due to the uncertainty that can be attributed to various aspects of soft robots. For example, system uncertainties could include unmodelled or neglected dynamics. Given that soft robots can have nearly infinite dimensions (infinite degrees of freedom), it is important to account for the error arising from the missing dimensions as our model must be finite dimensional. In addition to a reduction in dimensionality, the controller synthesis techniques discussed require a linear model of the nonlinear plant, introducing further inaccuracies. This source of model uncertainty can also be accounted for by using robust control techniques.

In addition to the innately complex dynamics of soft robots that will be neglected in the models, there may also be uncertainties due to the operating conditions. Given the flexibility and compliance in these systems, there is room for deviations from expected dynamics with changes in external factors, such as temperature, humidity (Liu et al., 2019), and externally applied forces (Rothemund et al., 2020). There may also be inconsistencies in either the materials used in making the compliant actuators or the processes used to develop them.

Provided the many sources of uncertainty present in the field of soft robotics, it is important to use appropriate measures to represent such uncertainty and ensure the system remains stable for all possible plants. This work focuses its uncertainty analyses on unstructured (complex) uncertainty, rather than parametric (real) uncertainty. We use Bode plots to model the unstructured uncertainty in our models with a single frequency-dependent weighting function. Similarly, the previously discussed $H_{\infty }$ norm 12 will be exploited again to help understand the maximum uncertainty allowable for the given plants and controllers.

We can lump the various sources of error in the model dynamics into a single complex perturbation which will simplify the analysis. The complex perturbation, denoted Δ , represents any matrix such that ${\left\|\Delta \right\|}_{\infty }\leq 1$ is satisfied and its dimensionality is compatible with the plant, G . It is important to note that lumping the uncertainty this way may lead to a conservative uncertainty estimate. There are various methods of modelling uncertainty to capture how the true system could vary from the nominal model. In this work, we use a multiplicative uncertainty model, visualized in the inner loop dynamics block diagram provided in Figure 2D. This is the preferred type of lumped uncertainty according to (Skogestad and Postlethwaite, 2005). The multiplicative weight, $W_{I}$ ,

$$|W_{I}\left(j\omega \right)|\geq l_{I}\left(\omega \right)\forall \omega ,$$ (23)

is selected to set an upper bound on the multiplicative uncertainty, $l_{I}$ ,

$$l_{I}\left(\omega \right)=\underset{G_{p}}{\max}|\frac{G_{p}\left(j\omega \right)-G\left(j\omega \right)}{G\left(j\omega \right)}|,$$ (24)

where G is the selected nominal plant, and $G_{p}$ is the set of possible plants. Additive uncertainty is demonstrated in the outer loop dynamics of this work and presented in the block diagram provided in Figure 2E. Input additive weight uncertainty, $W_{A}$ ,

$$|W_{A}\left(j\omega \right)|\geq l_{A}\left(\omega \right)\forall \omega ,$$ (25)

is selected to set an upper bound on the additive uncertainty, $l_{A}$ ,

$$l_{A}\left(\omega \right)=\underset{G_{p}}{\max}|G_{p}\left(j\omega \right)-G\left(j\omega \right)|.$$ (26)

After selecting the weight, $W_{I}$ or $W_{A}$ , to envelope the uncertainty, we can multiply it with the closed loop transfer function, T , to analyze the system’s response with uncertainty. Provided $|W_{I}T|<1$ or $|W_{A}T|<1\;\forall \;\omega$ in the frequency range of interest, then the system has achieved robust stability. To investigate the uncertainty present within the system and develop a controller for the nominal plant, we need an appropriate linear representation of its dynamics.

### 2.3 Dynamic mode decomposition with control and the Koopman operator theory

In order to make use of the linear systems and robust control theory mentioned above, it is important to derive linear models of the soft system. Dynamic mode decomposition (DMD) can be used to develop linear models of complex nonlinear dynamical systems with no knowledge of the plant *a priori* (Tu et al., 2014). DMD is an empirical method that is used to linearly approximate the system’s underlying dynamics. DMDc is an extension of DMD as it disambiguates between the system’s intrinsic dynamics and its response to external forcing (Proctor et al., 2016). Soft robots can benefit from data-driven modelling approaches given that their dynamics are difficult to derive from first principles. Furthermore, this empirical system identification tool enables the physical soft systems to be reconfigured without rederiving mathematical models each time.

DMDc is closely related to the Koopman operator theory in that it uses a linear operator to describe highly complex, nonlinear system dynamics. Given that it is not tractable to work with infinite dimensionalities, as the Koopman operator entails, we can instead work with a finite dimensional approximation of it. Therefore, extensions of DMDc are relevant in the field of soft robotics since they offer a means of reducing high-dimensional dynamic systems to tractable low-order linear models. This is possible by extending the dictionary of observables in the state space to help account for nonlinearities that appear in the dynamics (Bruder et al., 2021). Related methods include eDMD, mrDMD, and SINDy, which could provide more accurate linear models of the nonlinear soft systems. eDMD works by increasing the dictionary of observables associated with the system (Li et al., 2017). While mrDMD combines DMD with wavelet theory and adjusts time resolutions and windowing to help account for varied temporal activity within the system dynamics (Kutz et al., 2016). Lastly, SINDy helps identify a sparse basis for the dynamical system (Brunton et al., 2016).

The following steps describe how to develop a model using DMDc (Proctor et al., 2016). First, the inputs and outputs of the system are selected to be recorded and stacked into the control snapshot vector, $u_{k}$ , and the state snapshot vector, $x_{k}$ , respectively. The input signal is varied, and all of the signals are recorded at each time step, kΔt . The recorded control input and state vectors are concatenated into matrices,

$$X=\left[\begin{array}{cccc} x_{1} & x_{2} & \ldots & x_{\mathrm{m-1}} \end{array}\right],$$ (27)

$$ϒ=\left[\begin{array}{cccc} u_{1} & u_{2} & \ldots & u_{\mathrm{m-1}} \end{array}\right],$$ (28)

as well as the state snapshot matrix shifted by Δt ,

$$X^{\prime }=\left[\begin{array}{cccc} x_{2} & x_{3} & \ldots & x_{m} \end{array}\right].$$ (29)

The matrices, X and ϒ , are concatenated into a single matrix, Ω , containing all of the recorded information:

$$\Omega =\left[\begin{array}{c} X\\ ϒ \end{array}\right].$$ (30)

Similarly, the unknown dynamic and control input matrices are concatenated as well:

$$\bar{G}=\left[\begin{array}{cc} \bar{A} & \bar{B} \end{array}\right].$$ (31)

The linear dynamic equation expressed in Eq. 5 can be reformulated in the scope of the DMDc algorithm as

$$X^{\prime }=\bar{A}X+\bar{B}ϒ$$ (32)

and rewritten in terms of the stacked matrices,

$$X^{\prime }=\bar{G}\Omega .$$ (33)

Then, we perform a pseudoinverse ( † ) on Ω through a singular value decomposition (SVD):

$$\Omega \approx U\Sigma V^{*},$$ (34)

and thus, solve the expression:

$$\bar{G}=X^{\prime }\Omega^{†}.$$ (35)

Substituting the pseudoinverse of Ω into the equation above, we can evaluate

$$\bar{G}=X^{\prime }V\Sigma^{-1}U^{*}$$ (36)

and further calculate the approximate dynamic and input matrices,

$$\left[\begin{array}{cc} \bar{A} & \bar{B} \end{array}\right]\approx \left[\begin{array}{cc} X^{\prime }V\Sigma^{-1}U_{1}^{*} & X^{\prime }V\Sigma^{-1}U_{2}^{*} \end{array}\right],$$ (37)

with

$$U^{*}=\left[\begin{array}{cc} U_{1}^{*} & U_{2}^{*} \end{array}\right],$$ (38)

for which $U_{1}^{*}\;\epsilon \;R^{n\times p}$ and $U_{2}^{*}\;\epsilon \;R^{l\times p}$ .

## 3 HASEL actuators

For this work, we used HASEL actuators to operate the demonstrative soft actuator platform (Figure 1A). These actuators are ideal to demonstrate controller operation as they can exhibit high-speed actuation (Kellaris et al., 2018; Johnson et al., 2020) and precise control (Volchko et al., 2022). In addition, they can be adapted to many robotic systems due to their versatile design space which can enable a wide range of morphologies and actuation modes such as contraction, expansion, bending, and twisting (Acome et al., 2018; Kellaris et al., 2018; Mitchell et al., 2019). HASEL actuators operate using an electrohydraulic mechanism; this mechanism uses principles of both electrostatic and hydraulic actuation, which enables efficient operation, controllable output, and high-speed response.

HASEL actuators are comprised of three main components: polymer film, a liquid dielectric, and conductive electrodes. The thin polymer film is formed into a shell which is filled with the liquid dielectric to create an enclosed pouch. Opposing electrodes are placed on either side of the actuator over a portion of the pouch. When a voltage (typically several kilovolts) is applied, electrostatic forces cause the electrodes to progressively “zip” together, displacing the fluid between the electrodes to a different region in the pouch, causing shape change of the structure. Due to the inextensible nature of the polymer film, this causes linear contraction in the actuator (Kellaris et al., 2018).

Additionally, HASEL actuators are operable outside of a laboratory and, more specifically, suitable for driving untethered soft robotic systems, due to their energy and power density, low-power consumption, and customizability. Although these devices require a high voltage stimulus, input currents are typically less than 1 mA, resulting in peak input power of only watts. These actuators have an inherent catch-state, due to their capacitive energy storage, wherein an actuated state can be held while consuming only milliwatts or less of power. HASELs have been shown to consume 100 times less power than a comparable servo motor while holding a position (Kellaris et al., 2021). Myriad examples of miniaturized DC-DC power supplies can be utilized to step-up battery level voltages to the necessary high voltage inputs, making these actuators an attractive solution for compact, battery-powered soft robotic systems (Mitchell et al., 2019; Mitchell et al., 2022; Wang et al., 2023).

## 4 Robust control implementation

The benchtop system (Figure 1A), similar to that described in (Volchko et al., 2022), was designed to imitate the upper portion of the human arm, where the artificial biceps and triceps muscles work together to control the orientation of the system’s end effector. We present an overview of the system signals that are necessary for the control scheme developed in this work in Figure 1B. Charge signals, $c_{i}$ , are sent through two channels in the HASEL electronics to regulate actuator voltage. The voltage state of each channel, $V_{i}$ , dictates the length of the corresponding actuator. Lastly, this contraction, $\delta_{i}$ , of both muscles determines the overall orientation of the lever arm, θ .

The dimensions of the system configuration are shown in Figure 2A, and the experimental setup is depicted in Figure 2B. The robotic arm consisted of a carbon fiber lever arm attached to a stationary wooden column using a low friction radial bearing. The wooden column was anchored to a wooden platform to secure the system. A stack of five contracting actuators (Part No. C-5020-15-01-C-CCBC-50-140) were electrically and mechanically connected in parallel to form the biceps muscle, and a separate stack of five identical actuators were used for the triceps muscle. All HASEL actuators were based on the Peano-HASEL design described in Kellaris et al. (2018) and provided by Artimus Robotics. Their parameters can be found in the Supplementary Table S1.

The electronics package to drive actuation was also provided by Artimus Robotics (Part No. PS2-10-030-02). This power supply consisted of two independently addressable high voltage amplifiers. A Teensy 4.0 microcontroller was used to generate control signals, $c_{i}$ , for the high voltage amplifiers (0–3.3 V pulse width modulated signals with variable duty cycle) as well as receive analog information on high voltage output, $V_{i}$ . We attached rigid bodies to the system in various locations to track the displacement of the individual actuators, $\delta_{i}$ , and the orientation of the lever arm, θ . We 3D printed the rigid bodies and connected three retroreflective markers to each one. We secured the rigid bodies onto both ends of the muscles and attached one to the lever arm. We used seven OptiTrack motion tracking cameras (Figure 2B) and set the motion capture data acquisition rate to its maximum of 240 Hz to be streamed in real time.

The controller design process is comprised of the following individual procedures: system identification, model validation, controller synthesis, and uncertainty analysis. This is followed by controller simulation, implementation, and validation. The electrical dynamics were assumed to be significantly faster than the physical dynamics (Rothemund et al., 2020), and thus a cascaded control approach was used, as shown in Figures 2D,E. Additionally, this control scheme made the signals easier to isolate in order to analyze and study them.

The following methods, technologies, and software were used to execute closed loop control of the system described above. ROS was used to communicate with the multi-channel high power supply and the motion capture system, while running the data collection and controller scripts on a Linux OS. Arduino timer interrupts and ROS timers regulated the data acquisition and control rates. ROS data was timestamped in real time with respect to all ROS topics. All scripts can be found within the linked repository.

All data collection for the inner loop (Figure 2D) was performed at 2000 Hz. The control loop for the voltage dynamics was on board the microcontroller and set to run at 1,000 Hz. However, the communication protocol used to communicate between the microcontroller and the Linux machine over USB was limited to 200 Hz. Communication rates greater than 200 Hz became inconsistent due to the low priority of USB bulk transfers, which the Teensy 4.0 serial communication is built on, and therefore, resulted in instability. Thus, the physical dynamics (Figure 2E) were analyzed with a data collection rate of 200 Hz, and the outer loop was closed at 200 Hz.

Finally, MATLAB (MathWorks R2023), its robust toolbox, and its Simulink environment were used for all of the post processing, data analysis and controller synthesis work.

### 4.1 Voltage control

The power supply provided by Artimus is fundamentally a current controlled system, where the duty cycle of the pulse-width modulation (PWM) signal controls the amount of current supplied to the actuators. However, a voltage-controlled system is desirable, since voltage is an easily measured parameter that provides a direct analog to the physical state of the actuator (as the actuator performance is proportional to the applied voltage squared (Kellaris et al., 2018)). Thus, our control approach began with synthesizing a voltage controller to regulate the output voltage at each channel by adjusting the duty cycle of the applied PWM signals.

#### 4.1.1 System identification and model validation

To perform system identification on the inner most loop, visualized in Figure 2D, we chose to represent the system with inputs of duty cycles of the PWM signals that were sent to the power supply unit, $c_{i}$ , and output signals of recorded high voltage outputs, $V_{i}$ . While working with the nonlinear HASEL-actuated system, we required that the state of the system during data collection remained within tight operating ranges to enable linearization at each “linear regime”. We initially implemented a basic trial and error PID controller closed around voltage to ensure minimal and controlled deviations around an operating voltage. This sort of iterative approach is typical for systems with integral action in their dynamics and for systems that already have working, yet suboptimal, controllers (Landau and Rolland, 1994). While an initial guess and check PID controller proved to be sufficient for initial system identification efforts, it was apparent that the voltage response varied depending on the system’s operating point. Only 25% of the maximum duty cycle of PWM signals was used during testing to ensure the safety of the electronics and eliminate any catastrophic failures with the HASEL actuators.

**FIGURE 3:**
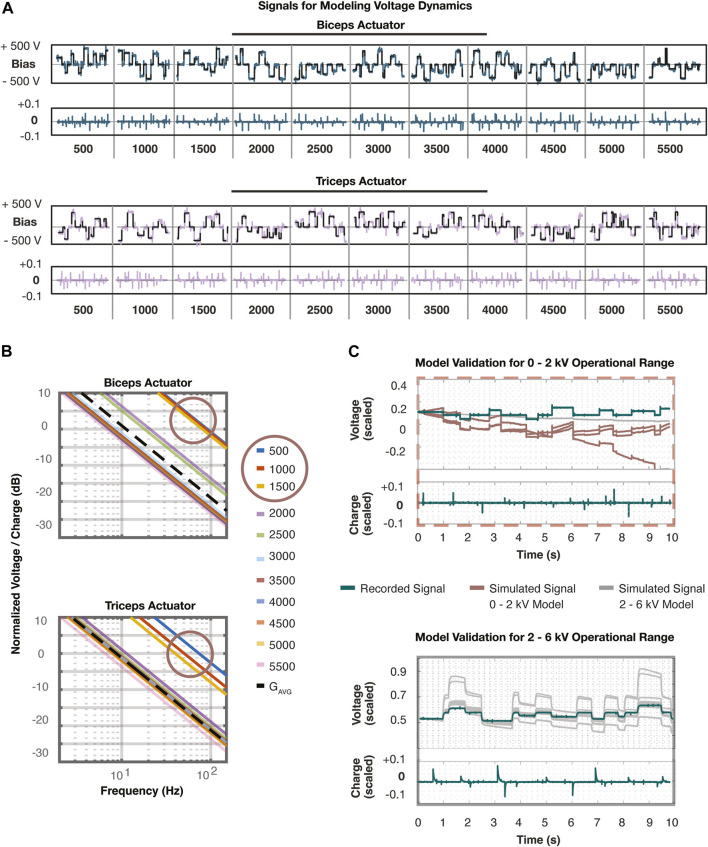
**(A)** These plots represent all data collected to identify linear models of the voltage dynamics. A reference voltage was sent to the initially implemented voltage controller to remain within small regions of the operational voltage range. A linear model was identified at every 500 V step from 500 to 5500 V for both the biceps and triceps muscles. The charge input was recorded and the voltage that followed was recorded. The vertical lines distinguish the segments of data corresponding to each voltage bias listed at the bottom. Each segment corresponds to 10 s of data collection. **(B)** The 22 transfer functions are plotted that were discovered using DMDc on each segment of data in **(A)**. Additionally, an estimate (black dashed line) of the average of these transfer functions is depicted. The two distinct regions of dynamics are apparent in this figure, where the linear models formed around the voltage biases 500–1500 V (circled) have a much higher magnitude on the Bode plot than the others. This demonstrated the need to break the system into two distinct regions and proceed by developing and analyzing one controller for each region. **(C)** The top plot demonstrates the validation of the voltage dynamics in the 0–2 kV range where the input and output data (teal) was recorded and compared against the simulated response of all linear models. The dark red lines denote the models within this range, while the grey lines represent the models in the 2–6 kV range. It is apparent these models demonstrate little response to the recorded input and appear as a nearly flat line. The bottom plot demonstrates the validation of the voltage dynamics in the 2–6 kV range where the input and output data (teal) was recorded and compared against the simulated response of all linear models. However, the linear models from the 0–2 kV range quickly deviated from the initial state and therefore were not plotted alongside.

To collect data for system identification, the closed loop system was sent a voltage reference profile around incremental voltage biases that ranged from 500 V to 5500 V. We selected multiple voltage biases due to the aforementioned variability in voltage responses depending on the operating point. The reference voltage profiles then varied from the initial biases by ± 100 − 400 V. The data collection process was carried out for both the biceps and triceps muscles to ensure we accounted for the uncertainty seen across all voltage dynamics within the system. Data was collected on the microcontroller at 2000 Hz for 10 seconds at each voltage bias. At the end of each segment, the voltage was sent over USB back to the Linux machine. The collected data is depicted in Figure 3A divided into 22 segments corresponding to the eleven voltage biases commanded to each of the muscles.

Before developing the linear models, all signals were normalized with respect to their maximum values prior to performing DMDc to help the interpretability of the Bode plots. The voltage signal was normalized with respect to the estimated maximum voltage of the power supply at 6000 V and the charge signal was normalized with respect to the maximum 12-bit resolution PWM signal duty cycle of 4096. Applying the DMDc algorithm following Eqs 27-38 to each segment of normalized data resulted in 22 distinct models of the voltage dynamics in the form Eq. 5. These 22 models represent the collection of possible plants, $G_{p}$ , referenced in Eq. 24. As demonstrated in the singular value plots in Figure 3B, three of the transfer functions from each actuator resulted in significantly higher gains. Therefore, the resultant voltage controller synthesis and analysis was split over two voltage domains, $V_{+}=$ 2000 − 6,000 V and $V_{-}=$ 0 − 2,000 V, to account for the nonlinear actuation response caused by an electrostatic pull-in instability (Acome et al., 2018). The nominal model of the voltage dynamics, $G_{V}$ , was determined in the frequency domain using an approximated average of all Bode plots. The eight slower plants from the biceps corresponding to the $V_{+}$ range resulted in the nominal transfer function, $G_{V_{b}}=70/s$ , while the corresponding transfer functions of the triceps actuators resulted in an averaged transfer function of $G_{V_{t}}=57/s$ . For the uncertainty analysis, we used the nominal plant, $G_{V+}\left(s\right)=70/s$ and synthesized a voltage control law for the muscles within the $V_{+}$ operating range. We repeated the above steps with the faster transfer functions to result in a final nominal plant of $G_{V-}\left(s\right)=445/s$ for voltage dynamics between 0 and 2,000 V. The plots in Figure 3C demonstrate the validity of these models in both operating regions of the voltage range. The true response is plotted alongside the simulated charge input to voltage output data.

#### 4.1.2 Controller synthesis

**FIGURE 4:**
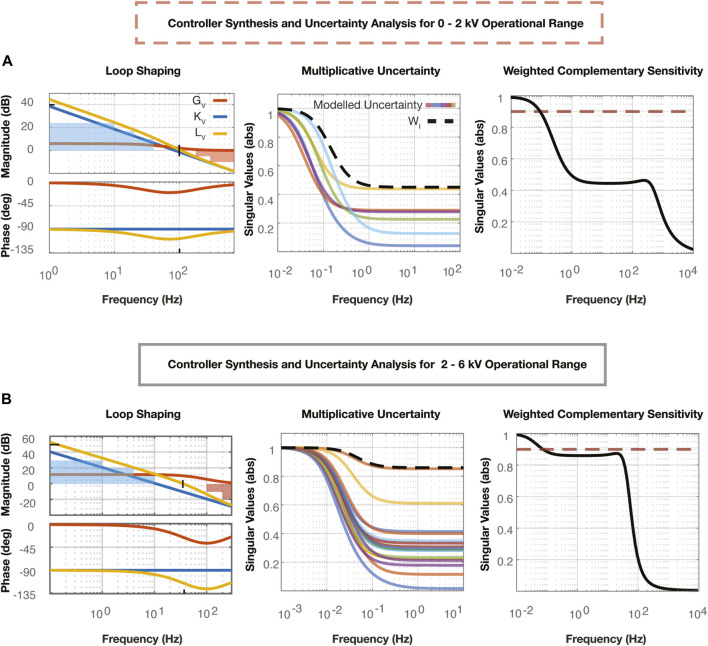
**(A)** The Loop Shaping plot represents the open loop shaping process used to synthesize the voltage controller in the 0–2 kV range. All three transfer functions are plotted, including the nominal plant, $G_{V}$ , the controller, $K_{V}$ , and the open loop, $L_{V}=G_{V}K_{V}$ . The two shapes plotted correspond to estimates for the closed loop performance of tracking performance (blue), and disturbance rejection (red). Information about the steady state error, overshoot, and system bandwidth estimates of the closed loop system are also pictured with the black hash marks. The Multiplicative Uncertainty plot depicts the modelled uncertainty, $l_{I}$ , which corresponds to the 6 transfer functions encircled in Fig.3B which represent the possible plants, $G_{p}$ , in the 0–2 kV operating range. The transfer function, $W_{I}$ , is developed and superimposed to create the upper bound on the multiplicative uncertainty, $l_{I}$ , as $\left|W_{I}\left(j\omega \right)\right|\geq l_{I}\left(\omega \right)\;\forall \;\omega$ . Weighted complementary uncertainty, $W_{I}T$ , is plotted, where T is the complementary sensitivity of the 0–2 kV closed loop system. The magnitude of this transfer function remains under the value 1, guaranteeing stability for all nonlinearities accounted for with this uncertainty analysis. The dashed line represents an absolute magnitude of 0.9 which is the upper limit we put on the transfer function, leaving room for other means of variation and uncertainty in the voltage dynamics loop. **(B)** The Loop Shaping plot represents the open loop shaping process used to synthesize the voltage controller in the 2–6 kV range with the same properties as that shown in **(A)**. The Multiplicative Uncertainty plot depicts the modelled uncertainty, $l_{I}$ , which corresponds to the other 16 transfer functions from Fig.3B which represent the possible plants, $G_{p}$ , in the 2–6 kV operational range. Weighted complementary uncertainty, $W_{I}T$ , is plotted, where T is the complementary sensitivity of the 2–6 kV system.

Following the validation of the models, we used loop shaping methods to develop a controller for closed loop voltage control following the process outlined in Åström and Murray (2021). The Bode plots labelled “Loop Shaping” in Figures 4A,B depict the transfer function of the nominal plant, G , the controller, K , and the open loop, L , defined in Eq. 8. These plots demonstrate high gain at low frequencies to satisfy steady state error and tracking performance requirements, and low gain at high frequencies to satisfy disturbance rejection performance requirements. The crossover frequency approximates the closed loop system bandwidth, and the phase margin of the system provides an estimate of the closed loop overshoot.

The resultant lag controller, $K_{V+}$ , suggests the following nominal closed loop performance metrics: 1) A steady state error of less than 1%. 2) Tracking performances of up to 1 Hz, 4 Hz, and 10 Hz with errors less than 3.2%, 10%, and 32% respectively. 3) A closed loop bandwidth over 35 Hz. 4) 3.1 times noise rejection at 100 Hz and 10 times noise rejection at 200 Hz. 5) A closed loop phase margin of 64°, corresponding to a maximum overshoot of roughly 7%. The resultant lag controller is expressed in the continuous time form as:

$$K_{V+}\left(s\right)=\frac{0.003168s+3.981}{0.003168s+1}.$$

The resultant lag controller, $K_{V-}$ suggests the following nominal closed loop performance metrics: 1) A steady state error of less than 1%. 2) Tracking performances of up to 10 Hz, 20 Hz, and 40 Hz with errors less than 6.4%, 15%, and 32% respectively. 3) A closed loop bandwidth over 100 Hz. 4) 1.8 times noise rejection at 180 Hz and 3.1 times noise rejection at 300 Hz. 5) A phase margin of 72° which corresponds to a maximum overshoot of roughly 3.8%. The resultant lag controller is expressed in the continuous time form as:

$$K_{V-}\left(s\right)=\frac{0.003176s+1.995}{0.003176s+1}.$$

#### 4.1.3 Uncertainty analysis

Uncertainty analysis methods integrating the lag controller were used to determine if these controllers would suffice for all possible plants, $G_{p}$ , determined from the experimental data. Figures 4A,B show the uncertainty analyses that were performed to ensure the controller would work for each linear model developed in the two operational voltage ranges, $V_{-}$ and $V_{+}$ , respectively. We employ multiplicative uncertainty following the block diagram for the inner loop, Figure 2D. The Bode plots for the multiplicative uncertainty models, $l_{I}$ , calculated using Eq. 24, are shown alongside an enveloping transfer function, $W_{I}$ , determined following Eq. 23. $W_{I}$ and the complementary sensitivity T , calculated following Eq. 9, are used to determine robust stability for the plant and controller. The weighted transfer function, $W_{I}T$ , must remain below a magnitude of one to ensure the closed loop system with controller, $K_{V}$ , remains stable among all uncertainties. The “Weighted Complementary Sensitivity” plots in Figures 4A,B therefore demonstrate that these controllers stabilize the full set of possible plants, $G_{p}$ .

#### 4.1.4 Controller simulation, implementation, and validation

**FIGURE 5:**
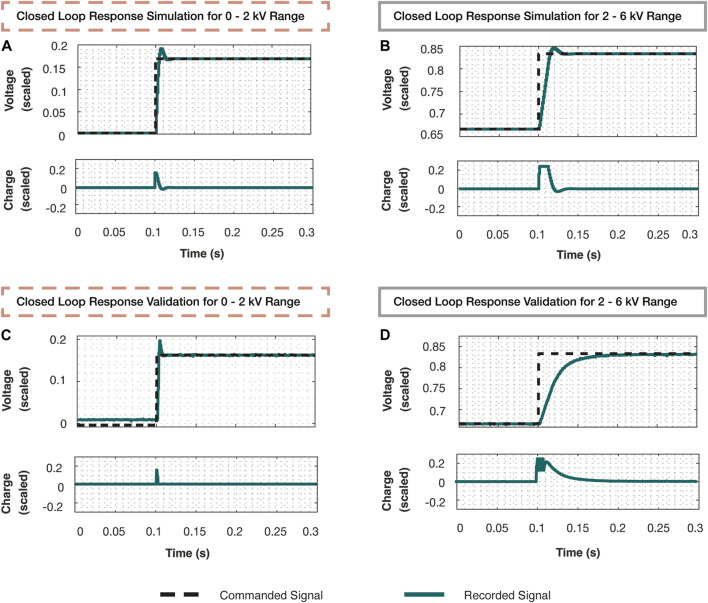
**(A)** The step response of the simulated closed loop system from 0 to 1 kV using the controller, $K_{V-}$ . **(B)** The step response of the simulated closed loop system from 4 to 5 kV using the controller, $K_{V+}$ . **(C)** The step response of the physical closed loop system from 0 to 1 kV which was implemented so the controller $K_{v–}$ would be used in the 0−2 kV operational range as it was scheduled based on the system’s state of voltage. **(D)** The step response of the physical closed loop system from 4−5 kV which was implemented so the controller $K_{V+}$ would be used in the 2−6 kV operational range as it was scheduled based on the system’s state of voltage. The limit set on the anti-windup compensator was 25% of the total charge allowed, and the charge can be seen hitting this limit in both the simulation **(B)** and the physical validation.

Before implementing the voltage controller on the physical system, a simulation was created in the Simulink environment, with additional measures to ensure that a discrete-time controller maintained similar performance to the continuous-time controller. The resulting closed loop simulations can be found in Figures 5A,B. The new lag compensators were discretized at 1,000 Hz using a zero-order hold and implemented into the system as

$${\bar{K}}_{V+}=\frac{z+0.07763}{z-0.7293}$$

and

$${\bar{K}}_{V-}=\frac{z-0.461}{z-0.7299}.$$

We implemented the gain scheduling approach based on the scheduling variable, voltage. If the recorded voltage signal was less than 2,000 V, ${\bar{K}}_{V-}$ would be used, while if the voltage was 2,000 V or above, ${\bar{K}}_{V+}$ would be used. In addition to the controller, an anti-windup compensator was added to the system around the minimum and maximum control outputs corresponding to 25% of the total PWM duty cycle to ensure the safety of the electronics. Given that the plant signals were scaled for controller synthesis, we implemented the controller with appropriately scaled corresponding signals. Finally, after implementing this controller in the firmware, we sent 1,000 V step commands to the closed loop system to validate the previously discussed performance. Performance metrics measured from these 0–1 kV and 4–5 kV step responses include 10%–90% rise times of 0.0013 s and 0.0416 s, 2% settling times of 0.008 and 0.163 s, and lastly overshoots of 22.1% and 0%, respectively, as shown in Figures 5C,D. As expected, the closed loop controller results in different performance conditions at differing voltage levels.

### 4.2 Position control

After the inner loop voltage controller was implemented, we synthesized the outer loop displacement controller. The first block, $f\left(\theta_{\text{REF}}\right)$ , in the block diagram (Figure 2E), translates the lever arm orientation into corresponding displacements of the actuators. The displacement commands, $\delta_{{\text{REF}}_{1}}$ , $\delta_{{\text{REF}}_{2}}$ were therefore:

$$\delta_{{\text{REF}}_{1}}=\sqrt{h_{1}^{2}+b_{1}^{2}-2h_{1}b_{1}\cos\left(\theta_{\text{INIT}}-\theta_{\text{REF}}\right)}$$ (39)

and

$$\delta_{{\text{REF}}_{2}}=\sqrt{h_{2}^{2}+b_{2}^{2}-2h_{2}b_{2}\cos\left(\theta_{\text{INIT}}+\theta_{\text{REF}}\right)}$$ (40)

with

$$\theta_{\text{INIT}}=\cos^{-1}\left(\frac{l^{2}-h_{1}^{2}-b_{1}^{2}}{-2h_{1}b_{1}}\right).$$ (41)

All variables are depicted in the system setup in Figure 2A and describe the system’s initial orientation, $\theta_{\text{INIT}}$ , commanded orientation, $\theta_{\text{REF}}$ , distances to biceps muscle anchor points, $h_{1}$ and $b_{1}$ , and distances to triceps muscle anchor points, $h_{2}$ and $b_{2}$ .

#### 4.2.1 System identification and model validation

To begin identifying the dynamics of the MIMO plant, G , we chose the input signals to our system to be the voltages, V , of each stack of HASELs and the output signals to be their respective displacements, δ . Before commanding the step inputs, we pretensioned the system to ensure that there was no slack in either the biceps or the triceps actuator to mitigate one source of nonlinear behavior. To pretension the system, a voltage bias was commanded to the biceps actuator, and the triceps contracted until a 0.1 mm extension occurred in the biceps. These two voltages were classified as the bias voltages.

**FIGURE 6:**
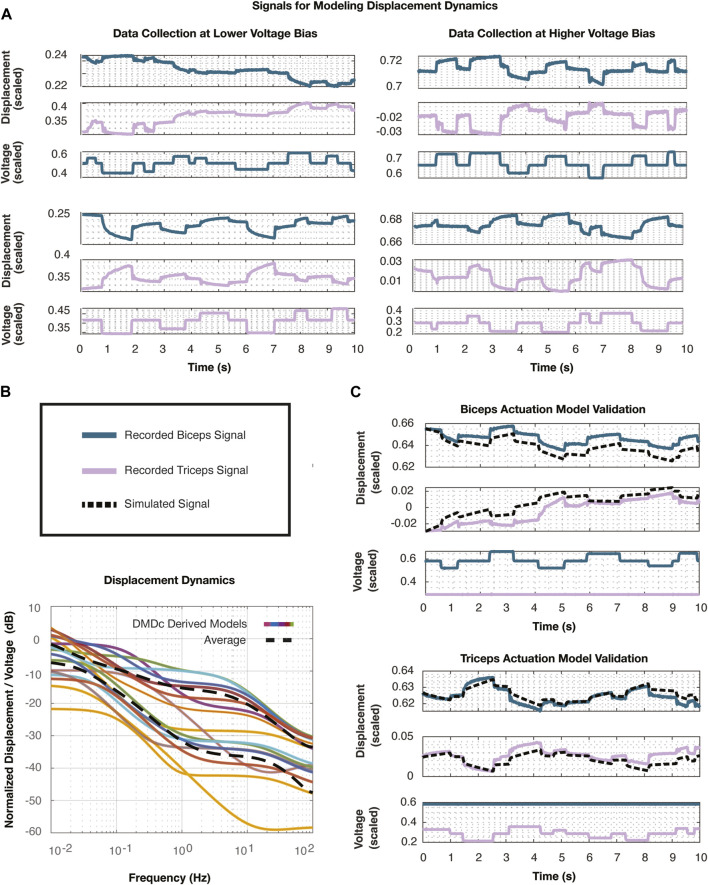
**(A)** These plots depict the signals collected to identify linear models of the actuator displacement dynamics. The system actuators were offset by a voltage bias, and two of these eight scenarios are plotted, for which the system is actuated by the biceps (top) and the triceps (bottom). **(B)** The singular value plots show all eight linear models developed using DMDc, each corresponding to a voltage bias that was set for data collection. The dashed line represents an approximate average of all of these transfer functions and was selected as the nominal plant for the controller synthesis and uncertainty analysis procedures. **(C)** Simulated step responses demonstrate voltage input to displacement output and validate the nominal models discovered with DMDc with respect to both biceps (top) and triceps (bottom) actuation.

Next, we commanded both muscles with random step inputs lasting 0.2–1.2 s and ranging in magnitudes of ±100−500 V around the voltage biases. This system identification data was recorded at each bias for 10 s of biceps actuation and 10 s of triceps actuation, and can be observed in Figure 6A.

We normalized all of the signals with respect to their maximum values to drive the intuition behind the following controller synthesis and uncertainty analysis procedures. The voltage was again scaled with respect to the rail voltage of 6,000 V and the displacement was normalized with respect to its maximum recorded displacement of 8 mm. Similar to the system identification approach of the voltage dynamics, we developed linear models following the DMDc algorithm in Eqs 27-38 from the various base voltages for the actuator displacement dynamics combining the biceps and triceps actuation profiles at each corresponding bias. Thus, we used eight distinct voltage biases to create eight distinct linear models where the state and input of the system were selected to be $x={\left[\begin{array}{cccccc} \delta_{1} & \delta_{2} & {\dot{\delta }}_{1} & {\dot{\delta }}_{2} & V_{1} & V_{2} \end{array}\right]}^{\prime }$ and $u={\left[\begin{array}{cc} V_{1} & V_{2} \end{array}\right]}^{\prime }$ , respectively, where subscripts 1 and 2 refer to the biceps and triceps muscles, respectively. The actuators’ velocities, ${\dot{\delta }}_{i}$ , were time derivatives of the collected displacement data smoothed using a Savitzky-Golay filter.

The resultant linear models are depicted in the singular value plot in Figure 6B. The transfer functions were averaged to result in the averaged linear model of the system (dashed line). We selected the DMDc model that most closely followed this average transfer function as the nominal plant in the following controller synthesis steps. The selected nominal model, $\bar{G}$ , discretized at 200 Hz, can be described with the following discrete-time matrices, following Eqs 5, 6.

$$\begin{array}{c} \bar{A}=\left[\begin{array}{cccccc} 1 & 0 & 0.0049 & -0.0002 & 0.0007 & -0.0009\\ 0 & 1 & 0.0002 & 0.0052 & -0.0019 & 0.0020\\ -0.0247 & 0.0004 & 0.5308 & -0.1366 & -4.9321 & 1.8180\\ -0.0084 & -0.0685 & -0.1894 & 0.6796 & 3.6924 & -5.0657\\ 0 & 0 & 0 & 0 & 0 & 0\\ 0.0001 & 0 & 0.0002 & 0.0005 & -0.0025 & 0.0019 \end{array}\right]\\ \;\bar{B}=\left[\begin{array}{cc} -0.0006 & 0.0009\\ 0.0019 & -0.002\\ 4.9730 & -1.8469\\ -3.6960 & 5.0963\\ 1 & 0\\ 0.0025 & 0.998 \end{array}\right]\;\\ \;\bar{C}=\left[\begin{array}{cccccc} 1 & 0 & 0 & 0 & 0 & 0\\ 0 & 1 & 0 & 0 & 0 & 0 \end{array}\right]\\ \;\bar{D}=\left[\begin{array}{cc} 0 & 0\\ 0 & 0 \end{array}\right]\text{.} \end{array}$$

We simulated the nominal plant using 20 s of validation data that was collected at the same voltage biases. This simulated data is compared with the empirical data in Figure 6C. The system was validated against both biceps and triceps input data, giving us confidence to proceed with the control design using the nominal plant, G .

#### 4.2.2 Controller synthesis

**FIGURE 7:**
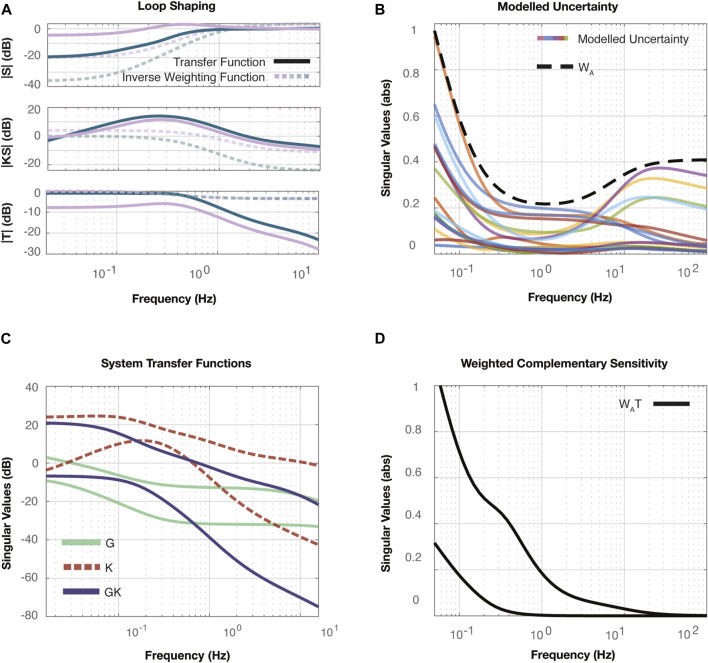
**(A)** The mixed synthesis results are shown with the three singular value plots. The top plot compares the resultant system’s sensitivity, S , and compares it to the inverse of the weighting, $W_{P}$ to help reach performance requirements, such as steady state error and tracking. The middle plot compares the closed loop transfer function, KS , to the inverse of the weighting, $W_{u}$ , to help minimize the control efforts from the system. And the last plot compares the complementary sensitivity, T , to the inverse of $W_{T}$ , to ensure the system does well in the presence of output disturbances, or noise. **(B)** The additive uncertainty, $l_{A}$ , is modelled in this singular value plot. The transfer function, $W_{A}$ is developed and superimposed to create the upper bound on the additive uncertainty, $l_{A}$ , as $\left|W_{A}\left(j\omega \right)\right|\geq l_{A}\left(\omega \right)\;\forall \;\omega$ . **(C)** The transfer functions plotted correspond to the plant, G , controller, $K_{\delta }$ and open loop, $L=GK_{\delta }$ to demonstrate the resultant “shape” of the system following $H_{\infty }$ controller synthesis. **(D)** The resultant weighted complementary sensitivity, $W_{A}T$ , is plotted. For our system to remain stable for all possible plants determined, it is critical that the magnitude of this transfer function remains under the value 1, which can be observed above $7\times 10^{-2}$ Hz.

After selecting a nominal plant, G , for the physical dynamics of the system, we used $H_{\infty }$ synthesis methods described in Section 2.1 to obtain an optimal controller, $K_{\delta }$ . The closed loop transfer function plots, S , KS , and T in Figure 7A demonstrate the transfer functions defined in Eqs 9, 13, 14. The weightings selected for the transfer functions correspond to system sensitivity to disturbances, control output response to reference commands, and the closed loop transfer function from reference signal to system output. We used the mixed sensitivity synthesis toolkit in the robust control package in MATLAB. The resulting weighted transfer functions,

$$\begin{array}{cc} & W_{p}=\left[\begin{array}{cc} \frac{s^{2}+12.31s+37.9}{s^{2}+1.87s+0.5831} & 0\\ 0 & \frac{s^{2}+9.234s+21.32}{1.5s^{2}+3.576s+2.132} \end{array}\right]\\ & W_{u}=\left[\begin{array}{cc} \frac{16s^{2}+96s+144}{s^{2}+24s+144} & 0\\ 0 & \frac{s^{2}+36.1s+90.25}{s^{2}+24s+144} \end{array}\right]\\ & W_{T}=\left[\begin{array}{cc} \frac{1.5s+3}{s+3} & 0\\ 0 & \frac{1.5s+3}{s+3} \end{array}\right], \end{array}$$

were specified to maintain a closed loop bandwidth of 0.8 and 0.6 Hz and provide nearly 0% steady state error in the biceps muscle. These weightings were adjusted following the approach outlined in Eqs 18, 19. We allowed for greater steady state error in the triceps muscle, to ensure that there was room for error in the measurements used in the geometric functions 39–41. We limited the control effort by selecting weighting functions, $w_{u_{i}}$ , above 0 dB to pull down KS . Lastly, we chose $W_{T}$ to remain at 0 dB and under at higher frequencies to ensure the closed loop response, T , provided a stable steady state response to the reference signal.

These specifications resulted in the $H_{\infty }$ controller, ${\bar{K}}_{\delta }$ , using the MATLAB command [K, CL, GAM] = mixsyn (G, WP, WU, WT), which applies the calculations from Eqs 20, 21. This controller was discretized at 200 Hz and is given in the as Supplementary Figure S1, structured according to Eqs 5, 6 with matrices, ${\bar{A}}_{\delta }$ ϵ $R^{12\times 12}$ , ${\bar{B}}_{\delta }\;\epsilon \;R^{12\times 2}$ , ${\bar{C}}_{\delta }\;\epsilon \;R^{2\times 12}$ , ${\bar{D}}_{\delta }\;\epsilon \;R^{2\times 2}$ . ${\bar{K}}_{\delta }$ resulted in $\gamma_{\text{min}}$ , defined in Eq. 22, of 9.894. We desired $\gamma_{\text{min}}$ to be close to a value of one to indicate that all of performance specifications are met. Initially, only S and T were weighted to determine a controller resulting in a closed loop system which resulted in $\gamma_{\text{min}}\;<$ 1. However, simulation indicated that this controller required too high of an input voltage to meet the specifications. Therefore, we included the weighted closed loop transfer function, $W_{u}KS$ , to reduce the control effort required of the physical system which resulted in a larger value of $\gamma_{\text{min}}$ . Figure 7C demonstrates how the controller affects the selected nominal plant.

#### 4.2.3 Uncertainty analysis

An uncertainty analysis was performed for the actuator displacement dynamics. However, due to the large uncertainties induced when attempting to use a multiplicative model for uncertainty, we resorted to a less restrictive uncertainty analysis, employing additive model uncertainty. The additive uncertainty structure can be found in Figure 2E. We calculated the uncertainty using $l_{A}$ defined in Eq. 26. The singular value plot in Figure 7B demonstrates the modelled uncertainty, $l_{A}$ , of all possible plants, $G_{P}$ , (solid lines). Additionally, this plot demonstrates the weighting, $W_{A}$ (dashed line), determined following Eq. 25, used to provide an upper limit on the modelled uncertainty. Finally, to conclude the uncertainty analysis, we plotted the weighted complementary sensitivity, $W_{A}T$ , in Figure 7D to ensure the system remained below an absolute magnitude of one to ensure stability for the span of uncertainty modelled in the frequency range of interest.

#### 4.2.4 Controller simulation, implementation, and validation

**FIGURE 8:**
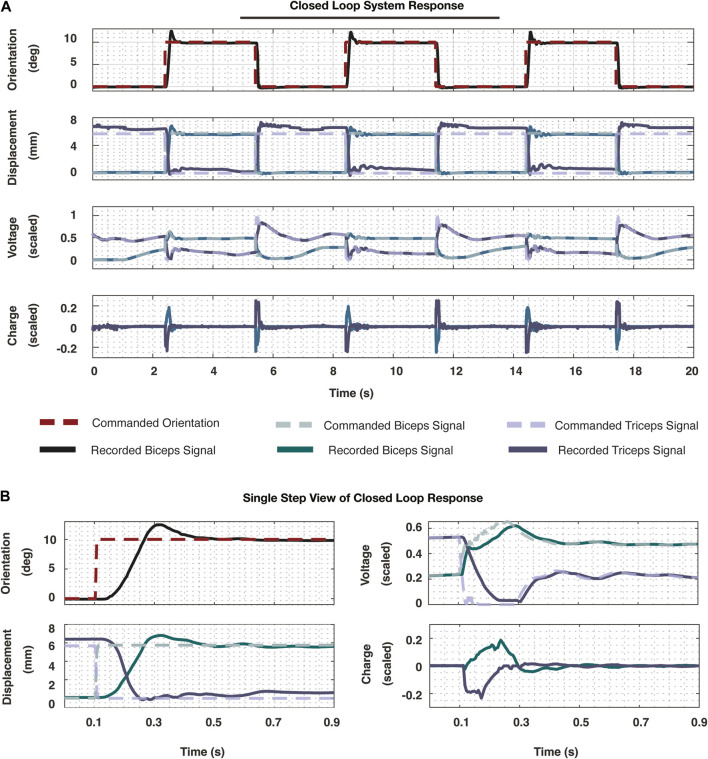
**(A)** The resultant signals are plotted from implementing the entire cascade controller developed in this work. The system was prescribed three step commands. The top plot demonstrates the reference orientation signal (dashed) and the recorded orientation (solid). These signals were recorded at 200 Hz. The following plot demonstrates the reference displacement signals that were commanded (dashed) and the recorded displacements (solid) of the two actuators. The subsequent plot further demonstrates the commanded voltage signal (dashed) and the measured voltage signal (solid). Finally, at the core of the system, the charge signals were plotted for each of the muscles. While the charge and voltage signals are scaled, the true measurements for displacement and orientation are plotted to highlight the scale of this work. **(B)** To truly visualize the response of the system, a single rising side of one step response is plotted, including all the signals represented in **(A)**. This demonstrates the speed of which the system was able to achieve steady state along with the other performance metrics noted in this work.

Prior to implementing the $H_{\infty }$ controller from the synthesis procedure, we simulated the closed loop system with the nominal plant in Simulink to ensure the system responded as expected. Following satisfactory simulation results, we implemented the controller on the physical system using various ROS nodes corresponding to the geometric function block, f(θ) , as well as the discretized $H_{\infty }$ controller, ${\bar{K}}_{\delta }$ , which was exported from MATLAB workspace to ROS files. Upon implementing the controller, we commanded step inputs to demonstrate the dynamic system response, shown in Figure 8A. The commanded orientation and orientation response are included in the top plot (Figure 8A) and used to assess the overall system performance. It should be noted that the accuracy of this control scheme relies on the accuracy of the measurements taken for actuator length, and anchor distances. Bypassing the function, f(θ) and directly measuring θ would allow for a more precise closed-loop response. However, we wanted to remove the dependence on the motion capture system and promote the implementation of self-sensing of individual actuator stacks for future work.

In observing a single step response in Figure 8B from this $H_{\infty }$ controller implementation, we demonstrate the following performance metrics. Resultant performance metrics measured from these step responses include 10%–90% rise times of 0.0877 s, 5% settling times of 0.8373 s, and lastly an overshoot of 24.75%. Further, we visualize the performance of the inner loop dynamics as well as the charge signals sent to the system in Figure 8B. These minimal charge signals demonstrate minimal wear on the system electronics.

## 5 Discussion and future directions

This paper outlines a novel framework for synthesizing robust control laws for soft robotic systems and demonstrates its implementation. We presented the first multivariable $H_{\infty }$ controller applied to a HASEL-actuated soft robot. Further, we advanced past work on similar soft systems by performing uncertainty analyses on the derived models to ensure the synthesized controllers stabilize the robot despite their evident nonlinear responses. We recommend this empirically-based framework as a starting point for similar soft robots due to its ability to achieve highly-performant closed loop dynamics as well as exhibit system stability in the presence of uncertainty.

Specific to the electrohydraulic actuators used in this work, we employed a cascade control architecture, allowing us to close the inner voltage control loop at a higher rate than the outer position control loop. Therefore, we developed the voltage control laws first. We determined simplified linear models of the voltage dynamics across the entire operating range using a data driven approach known as DMDc. The various models allowed us to address the variability within the nonlinear system’s full range of motion. Specifically, we identified two voltage regimes with distinct behaviors and correspondingly designed two separate lag controllers using loop shaping methods. We performed the uncertainty analyses for each regime by lumping the plant variability within a single complex perturbation. We implemented feedback control for each muscle using a gain scheduling procedure which resulted in closed loop rise times of 0.0013 and 0.0416 s, settling times of 0.008 and 0.163 s, and a stable response across the entire operational range.

To begin synthesizing a position controller, we identified a set of linear dynamic models to represent the motion of the mechanical system at different operating points. We selected a nominal model for each family of plants and employed $H_{\infty }$ controller synthesis techniques to optimize the nominal closed loop performance for steady state and system bandwidth. We assessed the outer loop uncertainty with an additive uncertainty model to ensure stability across the entire operating range. This control scheme was not designed to achieve the highest performance possible for the entire set of plants. Rather, the framework provided a simplified and systematic control solution that maintained a stable response throughout the entire range of motion of the mechanism. Even so, the closed loop system exhibited a rise time of 0.0877 s, and a settling time of 0.8373 s.

The success of this framework relies on properly selecting a linear model or set of linear models to represent the system. The linear models must appropriately estimate the complex dynamics of each unique system. More accurate models lend themselves to more appropriately designed controllers and more accurate uncertainty models. While the DMDc-derived models demonstrated substantial agreement to the system’s true response, the models did not capture the higher-frequency dynamics that were present. Extensions of DMD could better estimate physical system dynamics from empirical data. Some extensions include extended DMD (eDMD), multiresolution DMD (mrDMD) and Sparse Identification of Nonlinear Dynamics (SINDy) (Brunton et al., 2016; Kutz et al., 2016; Li et al., 2017). We suggest incorporating these methods when DMDc-derived models are not valid approximations of the physical plant.

Additionally, it would be advantageous to demonstrate this control methodology applied outside of a motion capture environment by utilizing integrated sensory feedback. A key advantage of HASEL actuators is their ability to simultaneously act as sensors and actuators (Acome et al., 2018; Ly et al., 2021). As deformable capacitors, HASEL actuators change capacitance as they actuate, and this capacitance change can be mapped to a change in the actuator displacement. Integrating capacitive self-sensing with the robust control methods elucidated in this work will broaden applications for electrohydraulic soft-actuated systems that operate outside of a laboratory environment. The developed framework can be implemented on untethered electrostatic systems powered by HASELs or DEAs, like those presented in Mitchell et al. (2019), Mitchell et al. (2022), Wang et al. (2023), Li et al. (2021) and Zhang et al. (2018).

These robust methods can extend past modelling a single system and can be used to determine the uncertainty across a range of actuators and capture the change in dynamics for a variety of actuator shapes, sizes, or materials. These robust methods could also be used to analyze the presence of extrinsic factors, such as loading or significant temperature changes. Using the methodology outlined in this article, we could work to understand stability regimes for a range of soft actuators or their varying environmental settings.

While we address robust stability and closed loop nominal performance individually, future work could focus on developing controllers that combine these two aspects to achieve robust performance of soft systems.

The framework outlined in this paper provides a methodology for applying linear control theory and robust stability measures to nonlinear soft robotic systems. Importantly, this method requires no knowledge of the system *a priori*. Implementing the measures of robustness outlined in this controller synthesis framework enables real-time, stable control of high-speed soft robotic systems, regardless of their means of sensing and actuation, inherent nonlinearities, or other uncertainties prevalent in the field of soft robotics. In order to unleash soft robots into the real world, we need to develop a generalized control framework that is just as adaptable and compliant as the physical systems.

## Data Availability

The datasets generated and analyzed for this study can be found in the Robust Control of Electrohydraulic Soft Robots github repository at https://github.com/avolchko/Robust_Control_of_Electrohydraulic_Soft_Robots.git.

## References

[B1] Acome E. Mitchell S. K. Morrissey T. G. Emmett M. B. Benjamin C. King M. (2018). Hydraulically amplified self-healing electrostatic actuators with muscle-like performance. Science 359, 61–65. 10.1126/science.aao6139 29302008

[B2] Åström K. J. Murray R. M. (2021). Feedback systems: an introduction for scientists and engineers. Princeton, NJ: Princeton University Press.

[B3] Bilodeau R. A. White E. L. Kramer R. K. (2015). “Monolithic fabrication of sensors and actuators in a soft robotic gripper,” in 2015 IEEE/RSJ international conference on intelligent robots and systems (IROS) (IEEE), 2324–2329.

[B4] Bruder D. Fu X. Gillespie R. B. Remy C. D. Vasudevan R. (2021). Data-driven control of soft robots using koopman operator theory. IEEE Trans. Robotics 37, 948–961. 10.1109/TRO.2020.3038693

[B5] Brunton S. L. Proctor J. L. Kutz J. N. (2016). Discovering governing equations from data by sparse identification of nonlinear dynamical systems. Proc. Natl. Acad. Sci. 113, 3932–3937. 10.1073/pnas.1517384113 27035946 PMC4839439

[B6] Chen C.-T. (2014). Linear system theory and design. in The Oxford Series in Electrical and Computer Engineering Series. Oxford University Press.

[B7] Du H. Li G. Sun J. Zhang Y. Bai Y. Qian C. (2023). A review of shape memory alloy artificial muscles in bionic applications. Smart Mater. Struct. 32, 103001. 10.1088/1361-665X/acf1e8

[B8] Dullerud G. E. Paganini F. (2013). A course in robust control theory: a convex approach, vol. 36. Springer Science & Business Media.

[B9] El-Atab N. Mishra R. B. Al-Modaf F. Joharji L. Alsharif A. A. Alamoudi H. (2020). Soft actuators for soft robotic applications: a review. Adv. Intell. Syst. 2, 2000128. 10.1002/aisy.202000128

[B10] Fuaad M. R. A. Hasan M. N. Muthalif A. G. A. Ali M. S. M. (2023). Electrostatic-hydraulic coupled soft actuator for micropump application. Smart Mater. Struct. 33, 015033. 10.1088/1361-665X/ad1428

[B11] Gu G.-Y. Zhu J. Zhu L.-M. Zhu X. (2017). A survey on dielectric elastomer actuators for soft robots. Bioinspiration Biomimetics 12, 011003. 10.1088/1748-3190/12/1/011003 28114111

[B12] Hegde C. Su J. Tan J. M. R. He K. Chen X. Magdassi S. (2023). Sensing in soft robotics. ACS Nano 17, 15277–15307. 10.1021/acsnano.3c04089 37530475 PMC10448757

[B13] Hess I. Musgrave P. (2023). “Nebula: a flexible, solid-state swimming robot enabled by hasel actuators,” in ASME 2023 conference on smart materials, adaptive structures and intelligent systems of smart materials, adaptive structures and intelligent systems), V001T06A004. 10.1115/SMASIS2023-110945

[B14] Johnson B. K. Sundaram V. Naris M. Acome E. Ly K. Correll N. (2020). Identification and control of a nonlinear soft actuator and sensor system. IEEE Robotics Automation Lett. 5, 3783–3790. 10.1109/LRA.2020.2982056

[B15] Kellaris N. Gopaluni Venkata V. Smith G. M. Mitchell S. K. Keplinger C. (2018). Peano-HASEL actuators: muscle-mimetic, electrohydraulic transducers that linearly contract on activation. Sci. Robotics 3, eaar3276. 10.1126/scirobotics.aar3276 33141696

[B16] Kellaris N. Rothemund P. Zeng Y. Mitchell S. K. Smith G. M. Jayaram K. (2021). Spider-inspired electrohydraulic actuators for fast, soft-actuated joints. Adv. Sci. 8, 2100916. 10.1002/advs.202100916 PMC8292915 34050720

[B17] Kim S. Laschi C. Trimmer B. (2013). Soft robotics: a bioinspired evolution in robotics. Trends Biotechnol. 31, 287–294. 10.1016/j.tibtech.2013.03.002 23582470

[B18] Kutz J. N. Fu X. Brunton S. L. (2016). Multiresolution dynamic mode decomposition. SIAM J. Appl. Dyn. Syst. 15, 713–735. 10.1137/15m1023543

[B19] Landau I. D. Rolland F. (1994). “An approach for closed loop system identification,” in Proceedings of 1994 33rd IEEE conference on decision and control (IEEE), 4, 4164–4169.

[B20] Li G. Chen X. Zhou F. Liang Y. Xiao Y. Cao X. (2021). Self-powered soft robot in the mariana trench. Nature 591, 66–71. 10.1038/s41586-020-03153-z 33658693

[B21] Li Q. Dietrich F. Bollt E. M. Kevrekidis I. G. (2017). Extended dynamic mode decomposition with dictionary learning: a data-driven adaptive spectral decomposition of the Koopman operator. Chaos Interdiscip. J. Nonlinear Sci. 27, 103111. 10.1063/1.4993854 29092410

[B22] Lipson H. (2014). Challenges and opportunities for design, simulation, and fabrication of soft robots. Soft Robot. 1, 21–27. 10.1089/soro.2013.0007

[B23] Liu X. Zhang J. Chen H. (2019). Ambient humidity altering electromechanical actuation of dielectric elastomers. Appl. Phys. Lett. 115, 184101. 10.1063/1.5126654

[B24] Ly K. Kellaris N. McMorris D. Johnson B. K. Acome E. Sundaram V. (2021). Miniaturized circuitry for capacitive self-sensing and closed-loop control of soft electrostatic transducers. Soft Robot. 8, 673–686. 10.1089/soro.2020.0048 33001742

[B25] Majidi C. (2019). Soft-matter engineering for soft robotics. Adv. Mater. Technol. 4, 1800477. 10.1002/admt.201800477

[B26] Mitchell S. K. Martin T. Keplinger C. (2022). A pocket-sized ten-channel high voltage power supply for soft electrostatic actuators. Adv. Mater. Technol. 7, 2101469. 10.1002/admt.202101469

[B27] Mitchell S. K. Wang X. Acome E. Martin T. Ly K. Kellaris N. (2019). An easy-to-implement toolkit to create versatile and high-performance HASEL actuators for untethered soft robots. Adv. Sci. 6, 1900178. 10.1002/advs.201900178 PMC6662077 31380206

[B28] Nguyen N. T. Sarwar M. S. Preston C. Le Goff A. Plesse C. Vidal F. (2019). Transparent stretchable capacitive touch sensor grid using ionic liquid electrodes. Extreme Mech. Lett. 33, 100574. 10.1016/j.eml.2019.100574

[B29] Niksefat N. Sepehri N. (2001). “Designing robust force control of hydraulic actuators despite system and environmental uncertainties,” in IEEE control systems magazine.

[B30] Pelrine R. Kornbluh R. Pei Q. Joseph J. (2000). High-speed electrically actuated elastomers with strain greater than 100%. Science 287, 836–839. 10.1126/science.287.5454.836 10657293

[B31] Proctor J. L. Brunton S. L. Kutz J. N. (2016). Dynamic mode decomposition with control. SIAM J. Appl. Dyn. Syst. 15, 142–161. 10.1137/15m1013857

[B32] Rothemund P. Kellaris N. Mitchell S. K. Acome E. Keplinger C. (2021). HASEL artificial muscles for a new generation of lifelike robots—recent progress and future opportunities. Adv. Mater. 33, 2003375. 10.1002/adma.202003375 PMC11469257 33166000

[B33] Rothemund P. Kirkman S. Keplinger C. (2020). Dynamics of electrohydraulic soft actuators. Proc. Natl. Acad. Sci. 117, 16207–16213. 10.1073/pnas.2006596117 32601189 PMC7368252

[B34] Rus D. Tolley M. T. (2015). Design, fabrication and control of soft robots. Nature 521, 467–475. 10.1038/nature14543 26017446

[B35] Schunk C. Pearson L. Acome E. Morrissey T. G. Correll N. Keplinger C. (2018). “System identification and closed-loop control of a hydraulically amplified self-healing electrostatic (HASEL) actuator,” in 2018 IEEE/RSJ international conference on intelligent robots and systems (IROS), 6417–6423. 10.1109/IROS.2018.8593797

[B36] Skogestad S. Postlethwaite I. (2005). Multivariable feedback control: analysis and design. Wiley.

[B37] Smerlas A. Walker D. Postlethwaite I. Strange M. Howitt J. Gubbels A. (2001). Evaluating H∞ controllers on the NRC Bell 205 fly-by-wire helicopter. Control Eng. Pract. 9, 1–10. 10.1016/s0967-0661(00)00088-5

[B38] Sundaram V. Ly K. Johnson B. K. Naris M. Anderson M. P. Humbert J. S. (2023). Embedded magnetic sensing for feedback control of soft HASEL actuators. IEEE Trans. Robotics 39, 808–822. 10.1109/TRO.2022.3200164

[B39] Thuruthel T. G. Ansari Y. Falotico E. Laschi C. (2018). Control strategies for soft robotic manipulators: a survey. Soft Robot. 5, 149–163. 10.1089/soro.2017.0007 29297756

[B40] Tu J. H. Rowley C. W. Luchtenburg D. M. Brunton S. L. Kutz J. N. (2014). On dynamic mode decomposition: theory and applications. J. Comput. Dyn. 1, 391–421. 10.3934/jcd.2014.1.391

[B41] Volchko A. Mitchell S. K. Morrissey T. G. Humbert J. S. (2022). “Model-based data-driven system identification and controller synthesis framework for precise control of SISO and MISO HASEL-powered robotic systems,” in 2022 IEEE 5th international conference on soft robotics (RoboSoft), 209–216. 10.1109/RoboSoft54090.2022.9762220

[B42] Walker J. Zidek T. Harbel C. Yoon S. Strickland F. S. Kumar S. (2020). Soft robotics: a review of recent developments of pneumatic soft actuators. Actuators 9, 3. 10.3390/act9010003

[B43] Wang T. Joo H.-J. Song S. Hu W. Keplinger C. Sitti M. (2023). A versatile jellyfish-like robotic platform for effective underwater propulsion and manipulation. Sci. Adv. 9, eadg0292. 10.1126/sciadv.adg0292 37043565 PMC10096580

[B44] Zhang H. Zhou Y. Dai M. Zhang Z. (2018). A novel flying robot system driven by dielectric elastomer balloon actuators. J. Intelligent Material Syst. Struct. 29, 2522–2527. 10.1177/1045389X18770879

[B45] Zhao H. O’Brien K. Li S. Shepherd R. F. (2016). Optoelectronically innervated soft prosthetic hand via stretchable optical waveguides. Sci. Robotics 1, eaai7529. 10.1126/scirobotics.aai7529 33157858

