# Supplementary material for: Robust control of electrohydraulic soft robots
Source: Front Robot AI. 2024 Aug 2;11:1333837. doi: 10.3389/frobt.2024.1333837 (PMC11327443; doi:10.3389/frobt.2024.1333837)
Supplement: Supplementary file 1 [file DataSheet1.pdf]

Supplementary Material

$$A = \begin{bmatrix}
1.00 & 0 & 0 & 0 & 0 & 0 & 0 & 0 & 0 & 0 & 0 & 0 \\
0 & 0.9994 & 0 & 0 & 0 & 0 & 0 & 0 & 0 & 0 & 0 & 0 \\
0.9061 & 0.7613 & 0.8152 & -0.1005 & 0 & 0 & -8.0025 & -6.0531 & -0.0571 & -0.0680 & 0.0354 & 0.2364 \\
0.4932 & 0.4354 & -0.0954 & 0.9332 & 0 & 0 & -4.4188 & -3.4289 & -0.0309 & -0.0392 & 0.0105 & 0.1399 \\
-0.0013 & -0.0011 & 0.0002 & 0.0002 & 0 & 0 & 0.0368 & 0.0084 & 0.0002 & 0.0001 & 0.0001 & 0.0004 \\
0.0006 & 0.0004 & 0 & -0.0002 & 0 & 0 & -0.0049 & 0.0221 & 0 & 0 & -0.0001 & 0.0003 \\
0.0546 & 0.0458 & -0.0084 & -0.0064 & 0 & 0 & 0.5178 & -0.3647 & 0.0013 & -0.0044 & -0.0020 & 0.0149 \\
0.0056 & 0.0054 & -0.0016 & 0.0007 & 0 & 0 & -0.0513 & 0.9582 & -0.0003 & 0.0046 & 0.0008 & -0.001 \\
32.9951 & 27.2587 & -4.4119 & -4.3991 & -0.0001 & -0.0004 & -290.044 & -217.429 & -1.5391 & -2.559 & -2.4029 & 9.9374 \\
-10.827 & -7.9633 & 0.4835 & 3.1588 & -0.0007 & 0.0009 & 92.2381 & 65.0255 & 0.5135 & 1.3806 & 2.3164 & -6.374 \\
9.6290 & 8.0854 & -1.4284 & -1.0663 & 0.0001 & -0.0001 & -85.0270 & -64.2887 & -0.6071 & -0.7226 & 0.5139 & 2.5102 \\
5.2483 & 4.6404 & -1.0137 & -0.1763 & -0.0001 & 0.0002 & -47.0397 & -36.5307 & -0.3287 & -0.4177 & 0.1076 & 1.6282
\end{bmatrix}
\quad
C = \begin{bmatrix}
0.0200 & 0 \\
0 & 0.0200 \\
0.0096 & 0.0080 \\
0.0052 & 0.0046 \\
0 & 0 \\
0 & 0 \\
-0.0002 & -0.0002 \\
0.0002 & 0.0001 \\
0.5589 & 0.4642 \\
-0.1227 & -0.0873 \\
0.1362 & 0.1145 \\
0.0744 & 0.0656
\end{bmatrix}$$

$$B = \begin{bmatrix}
13.2813 & 11.1818 & -2.0671 & -1.4785 & 0.0143 & -0.0103 & -117.3765 & -88.859 & -0.8358 & -0.9981 & 0.5089 & 3.4726 \\
7.2406 & 6.3600 & -1.4015 & -0.3335 & -0.0057 & 0.0156 & -64.7763 & -50.1439 & -0.4533 & -0.5716 & 0.1672 & 2.0336
\end{bmatrix}
\quad
D = \begin{bmatrix}
0 & 0 \\
0 & 0
\end{bmatrix}$$

Figure S1. H_∞ controller, $\bar{K}_\delta$, discretized at 200 Hz, used for displacement control of HASEL muscles in benchtop system.

Table S1. The following table highlights key parameters of the HASEL actuators that were used in this work. The full documentation of these HASELs can be found at Artimus Robotics, Part No. C-5020-15-01-C-CCBC-50-140.

HASEL Actuator Paramaters	
Actuation Type	Contracting
Series	C-Series
Model Number	C-5020
Actuator Stack Quantity	1
Pouches per Actuator	15
Mounting	Thin Symmetric Mounts
Weight	37 g
Dimensions (rest)	60 X 368 X 1.5 mm
Voltage Range	2 kV - 8 kV
Free Stroke	18 mm (@8 kV)
